# Correction to: Two serial filters control P2X7 cation selectivity, Ser342 in the central pore and lateral acidic residues at the cytoplasmic interface

**DOI:** 10.1093/pnasnexus/pgae460

**Published:** 2024-10-23

This is a correction to: Fritz Markwardt, Eike Christian Schön, Mihaela Raycheva, Aparna Malisetty, Sanaria Hawro Yakoob, Malte Berthold, Günther Schmalzing, Two serial filters control P2X7 cation selectivity, Ser342 in the central pore and lateral acidic residues at the cytoplasmic interface, *PNAS Nexus*, Volume 3, Issue 9, September 2024, pgae349, https://doi.org/10.1093/pnasnexus/pgae349

The author Mihaela Raycheva's name was inadvertently published as “Michaela Raycheva”. This error has been corrected.

