# Supplementary material for: Two serial filters control P2X7 cation selectivity, Ser342 in the central pore and lateral acidic residues at the cytoplasmic interface
Source: PNAS Nexus. 2024 Aug 23;3(9):pgae349. doi: 10.1093/pnasnexus/pgae349 (PMC11388005; doi:10.1093/pnasnexus/pgae349)
Supplement: pgae349_Supplementary_Data [file pgae349_supplementary_data.zip › PNASNEXUS-PNASNEXUS-2024-00360-TR-s01.docx]

**Supplementary Figures**


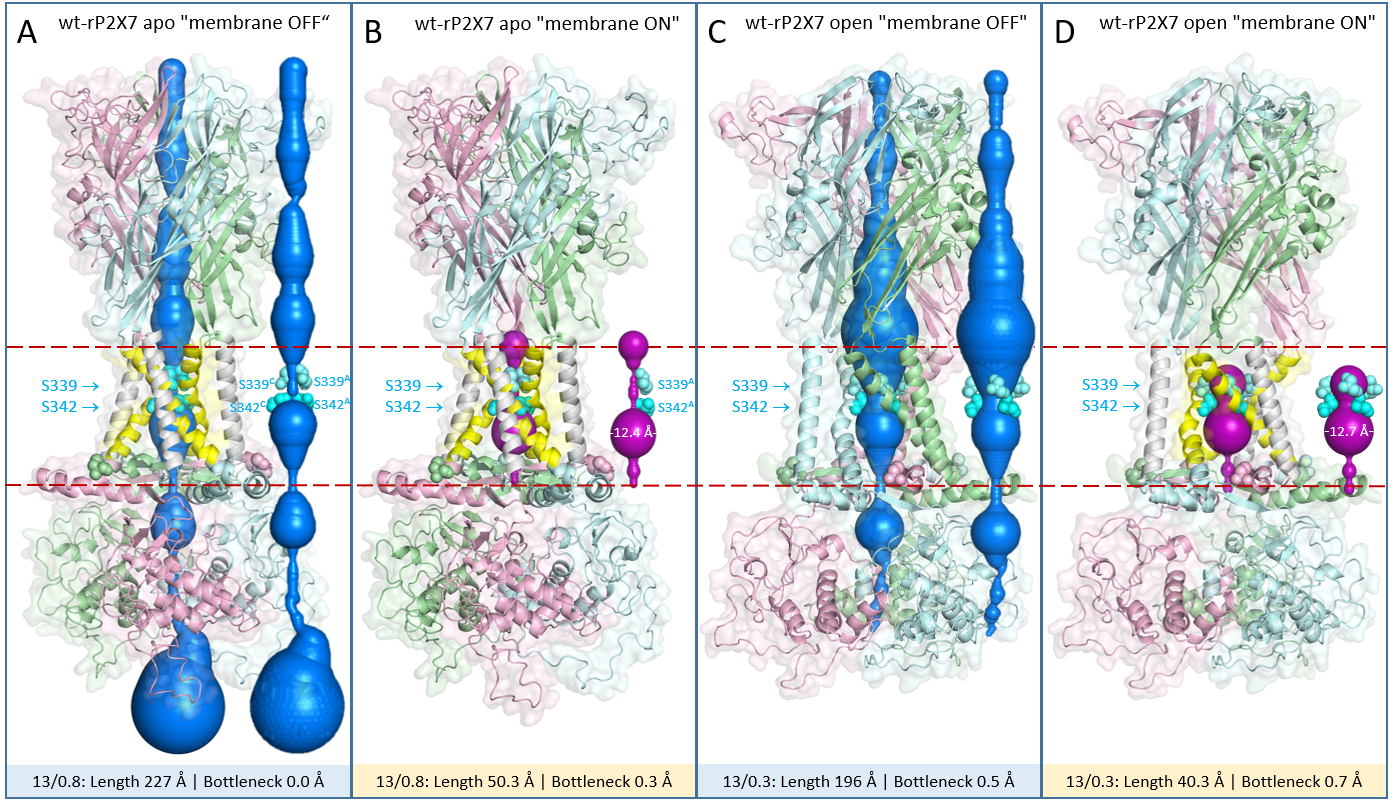


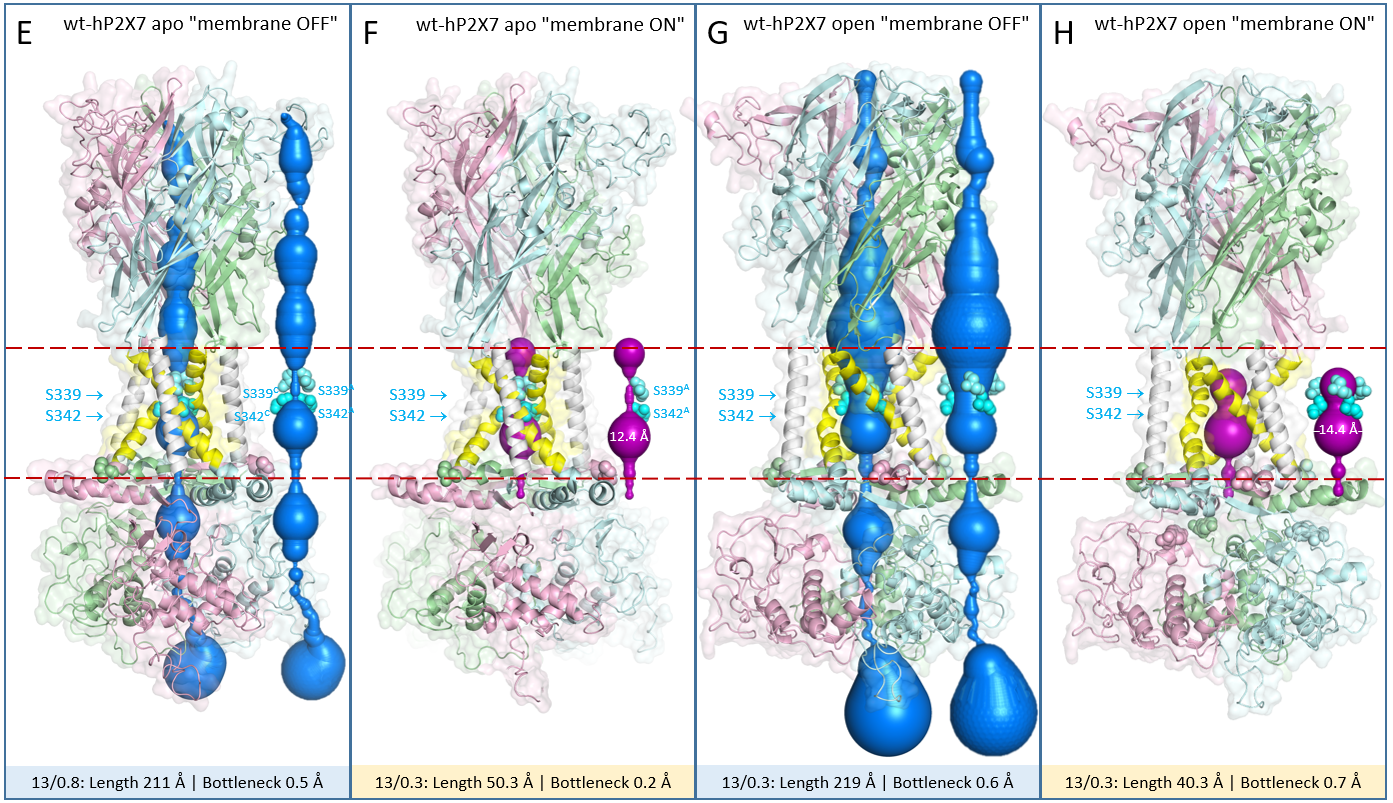


***SI Appendix*, Fig. S1. Pore structures of rP2X7R and hP2X7R calculated with the MOLEonline program without and with consideration of the position of the plasma membrane**. Shown are side views based on the cryo-EM structure of rP2X7R (A-D) and the derived SWISS homology model of hP2X7R (E-H). The calculated pore structures are also shown without the surrounding protein to visualize details of the bottlenecks. When one or two serine residues are omitted for better visibility in A,B and E,F, the serine residues shown are labeled with their residue numbers and the corresponding polypeptide chain as superscript. The predicted outer and cytoplasmic borders of the plasma membrane are indicated by the dashed red lines. The figure was generated with PyMOL.


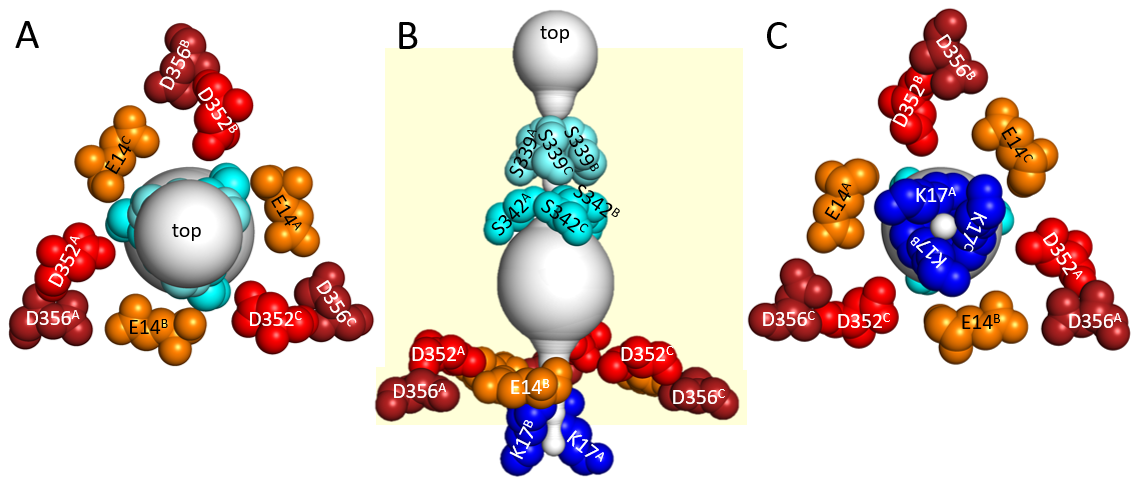


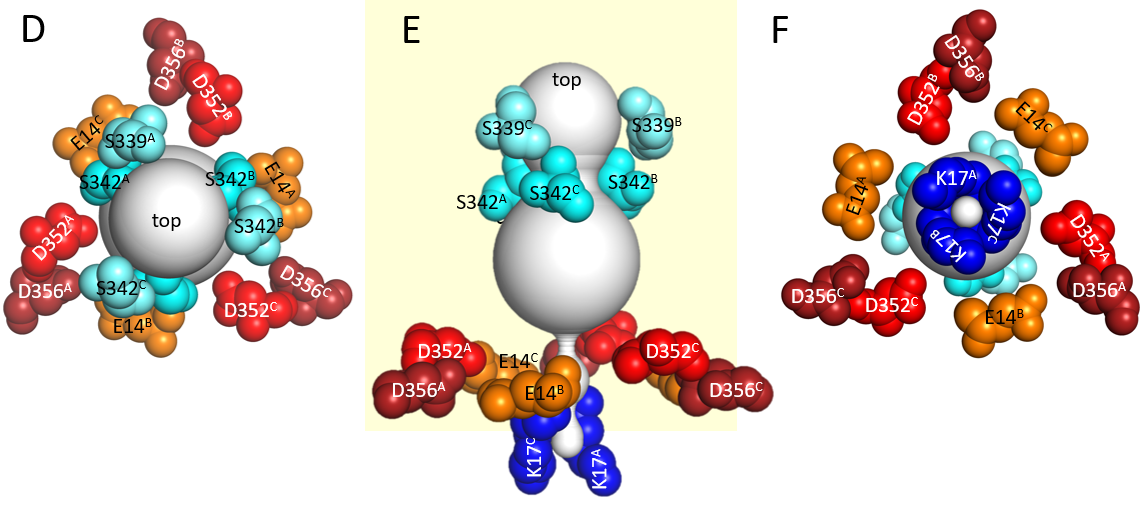


***SI Appendix*, Fig. S2. Location of acidic and basic residues at the cytoplasmic interface of hP2X7R^wt^ relative to the channel pore**. Shown are top (A,D), side (B,E) and bottom (C,F) views of the closed (A-C) and open (D-F) pores (in light gray) of hP2X7R as predicted by the MOLEonline program in the "membrane ON" configuration. The top views show the arrangement of the nine acidic residues, three from each subunit, in an isosceles triangular structure. The membrane position as predicted by the OMP (The Orientations of Proteins in Membranes) database is highlighted in light yellow (B,E). In both the closed and open states, the single exit to the cytoplasm predicted by MOLEonline, formed by three circularly arranged K17 residues, is far too narrow to allow ions to exit from the central vestibule above. The intracellular exit experimentally documented in the present work is lateral to the major axis of the hP2X7R channel through three pores, one per subunit interface, each lined by E14 and D352/D356 from adjacent subunits. The figure was generated using PyMOL.


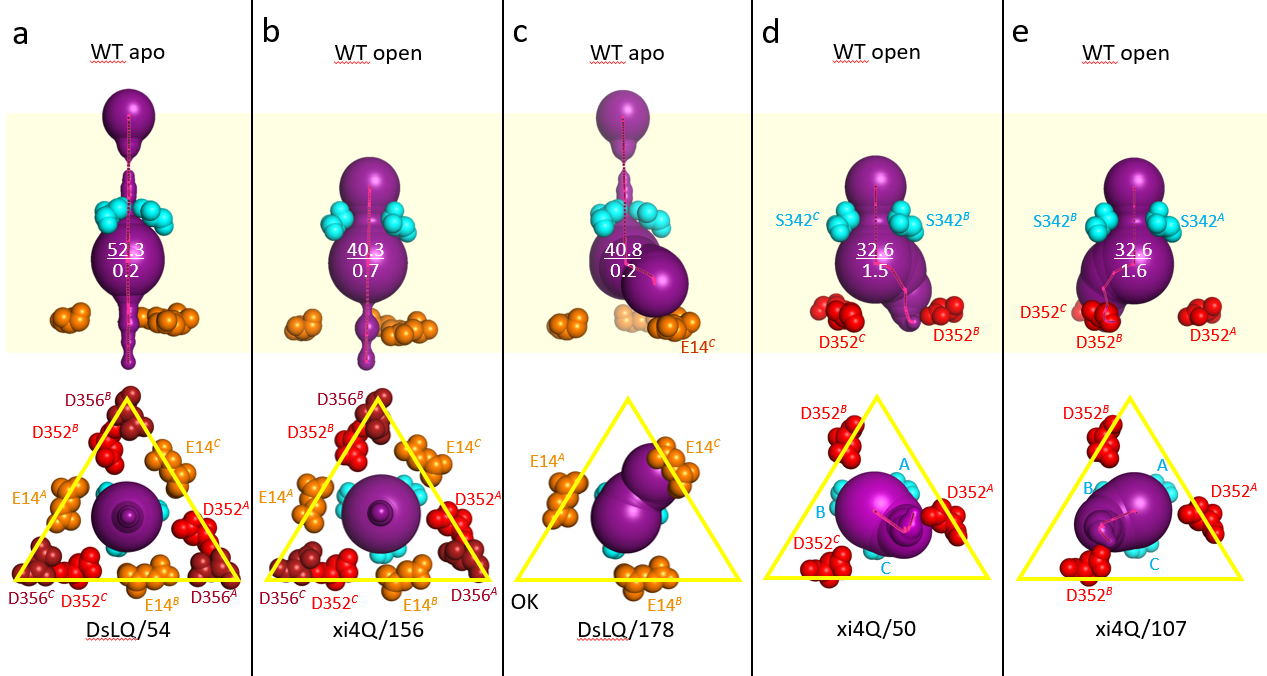


***SI Appendix*, Fig. 3. Lateral pores are randomly detected in the homology-modeled hP2X7R by MOLEonline**. Closed and open hP2X7R^wt^ structures were repeatedly analyzed with identical or varying settings of the MOLEonline software in the "Membrane ON" configuration. A,B represent the prevailing results in the side and bottom views already shown in Fig. 1. The side view shows only the positions of the E14 residues, while the bottom view shows all nine acidic residues within the isosceles triangle. C-E show examples obtained with identical MOLEonline settings that, in addition to the consistently modeled large vestibule, have a modeled lateral pathway flanked by one of the critical acidic residues, E14^B^ (C), D352^A^ (D), and D352^B^ (E). Serine 342 residues are shown in cyan. The numbers at the bottom are part of the bookmarks under which the specified data can be reopened. The lateral pores discovered in C-E do not increase the diameter of the large cavity below S342. This suggests that the lateral pores only branch below the equator of the cavity. The figure was generated using PyMOL.

Repetitive MOLEonline calculations (60 each) with identical default settings revealed lateral paths at different frequencies depending on the hP2X7R variants studied: about 15% for hP2X7R^wt^, 38% for hP2X7R^E14K^, 33% for hP2X7R^S342K^, 100% for hP2X7R^D352K^, and 12% for hP2X7R^D356K^.


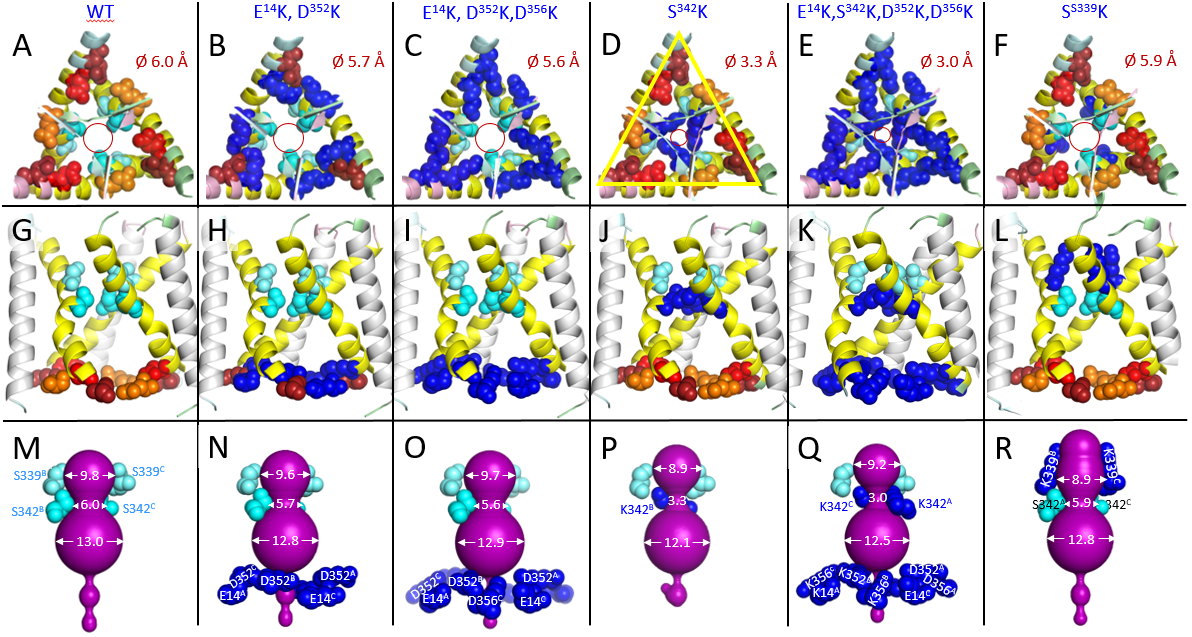


***SI Appendix*, Fig. S4. Pore size at the level of gating residue S342 is affected by lysine substitution of S342, but not by lysine substitution of acidic residues in the isosceles triangle at the cytoplasmic interface**. A-F show the top and G-L the corresponding side views of the membrane-embedded region of the indicated SWISS homology-modeled open hP2X7R constructs. M-R show the corresponding pore structure (in purple) as predicted by MOLEonline. The indicated diameters (in Å) of the upper and lower vestibules and the pores at S342 were graphically derived from the MOLEonline-predicted channel profiles. Orange, red and firebrick colored residues represent E14, D352 and D356, blue colored residues represent lysines. All mutants are functional except S339K (far right row), which is not functional when mutated alone or in combination. The highly distorted shape of the upper vestibule (seen in R) may prevent extracellular cations from entering the central pore, but this was not further investigated and is therefore speculative.


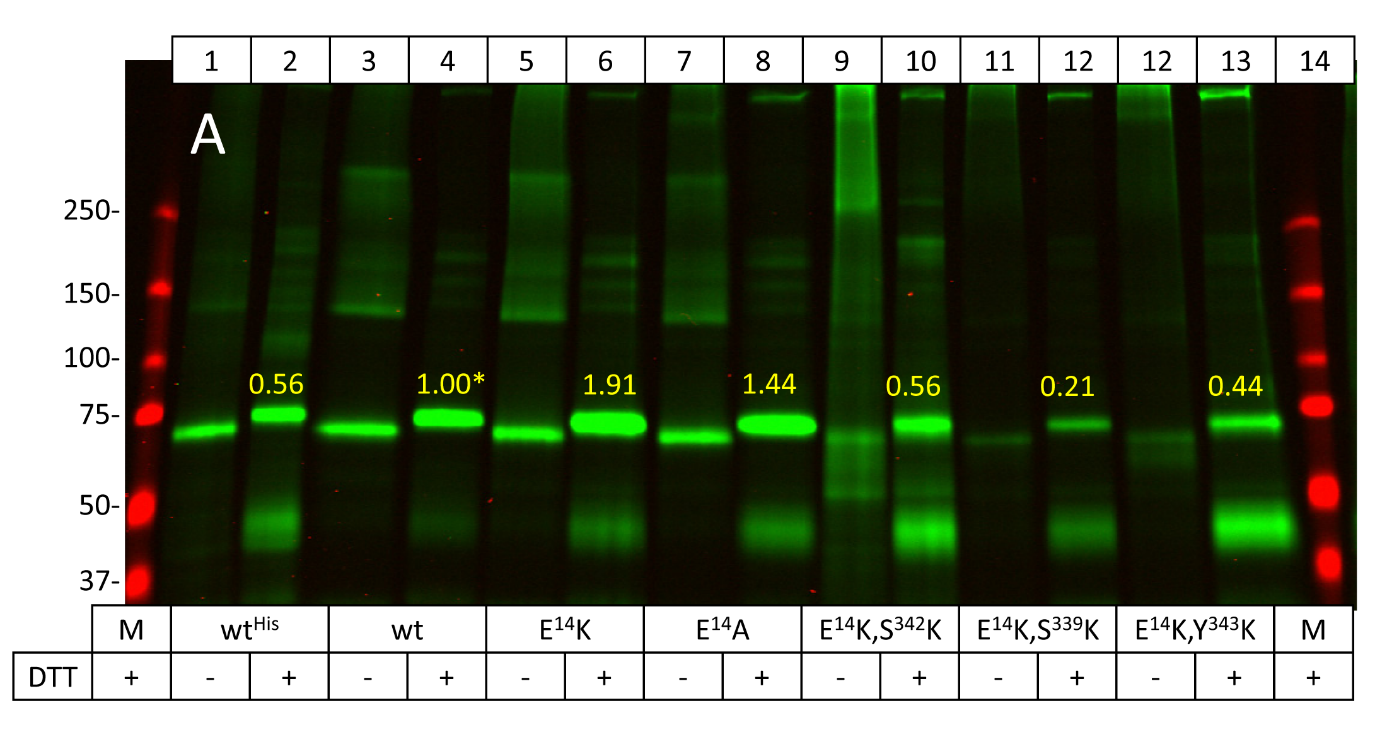


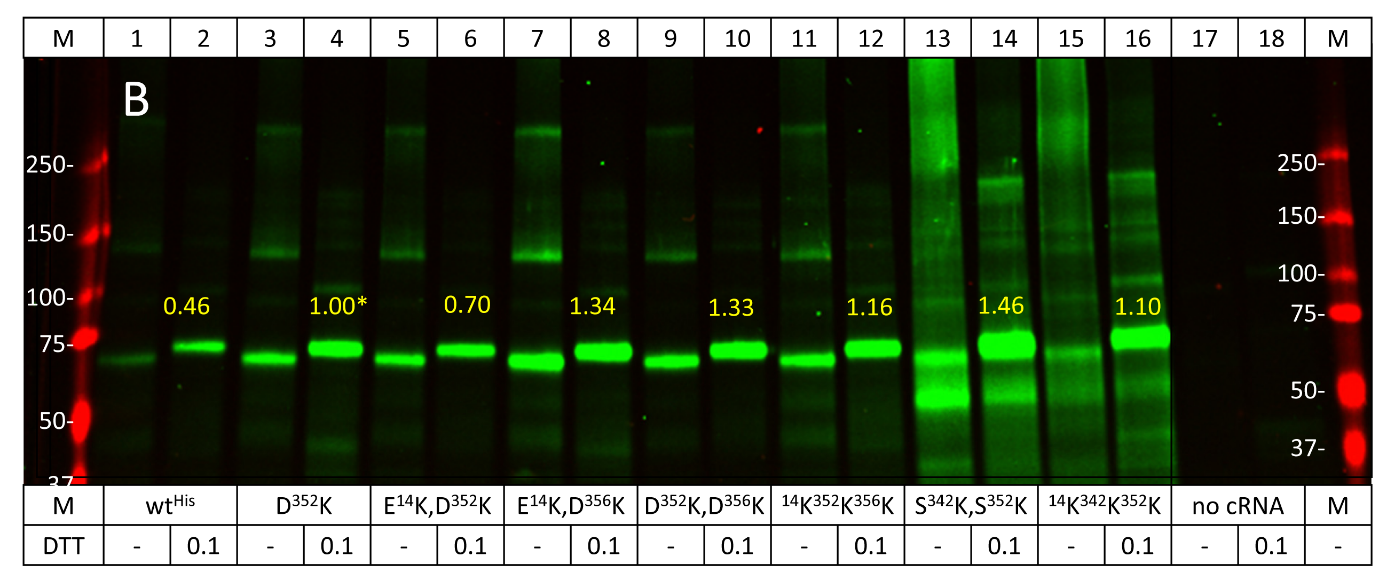


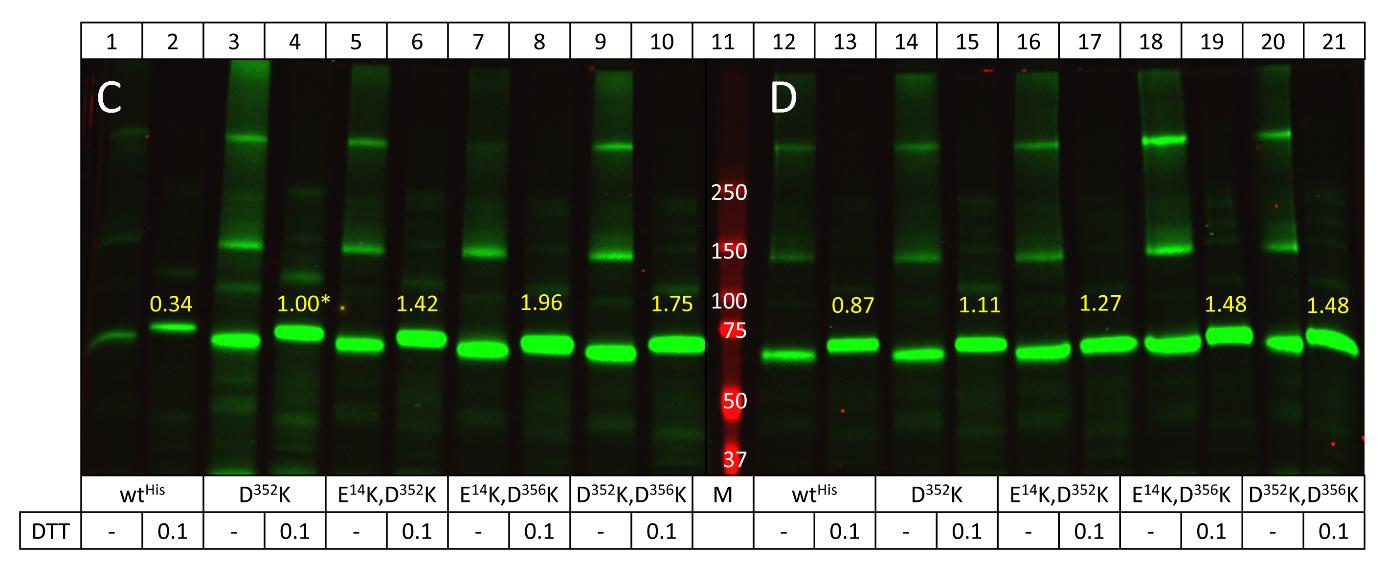


***SI Appendix*, Fig. S5. Plasma membrane expression of hP2X7R constructs in *X. laevis* oocytes.** The indicated oocyte-expressed hP2X7R constructs with C-terminal double-strepII tag (10-12 oocytes per sample) were labeled with the membrane-impermeant fluorescent dye IR800, solubilized with digitonin, affinity-purified on Strep-Tactin resin, and resolved by SDS-PAGE in non-reduced and DTT-reduced form as indicated, followed by fluorescence scanning and quantification using IMAGE Lab software (Bio-Rad). The equivalent of ~ one oocyte (= 10 µl of eluate) was applied per lane. Yellow numbers indicate fluorescence normalized to that of the asterisked band. Expression varied by a factor of up to 2 from construct to construct within individual experiments, with no clear evidence that any of the single mutations or combinations of mutations (up to 3 in B, lanes 11-12) strongly reduced surface expression. The only clear and reproducible reduction in plasma membrane expression (and function (Klapperstück et al 2001) was observed when the otherwise wt hP2X7R carried a C-terminal His tag (see lanes 1-2 in A, B and C and lanes 12-13 in D).

*M. Klapperstück, C. Büttner, G. Schmalzing, F. Markwardt*. Functional evidence of distinct ATP activation sites at the human P2X7 receptor. *J Physiol* **534**, 25-35 (2001).


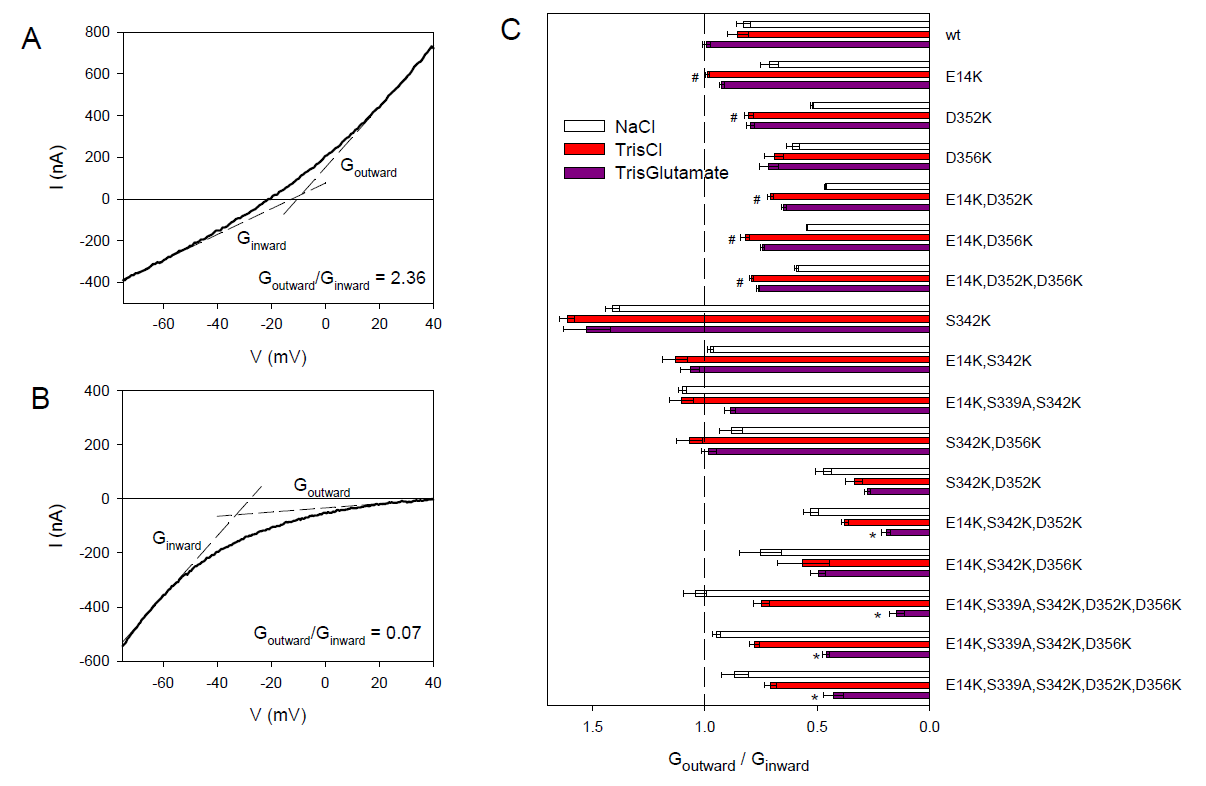
***SI Appendix*, Fig. S6. Rectification of hP2X7R^wt^ and mutants carrying 1-4 D/E to K mutations**. A and B, examples of (A) outward rectification (hP2X7^S342K^ in Tris^+^Cl^-^) and (B) inward rectification (hP2X7R^E14K,S342K,D352K^ in Tris^+^Glu^-^). The rectification indices are shown in the figures. C, Statistics of rectification index G_outward_/G_inward_ for the hP2X7R constructs studied. Symbol # indicates a significant change in rectification when Na^+^Cl^-^ was replaced by Tris^+^Cl^-^. Symbol * indicates a significant decrease in rectification index when Tris^+^Cl^-^ was replaced by Tris^+^Glu^-^. Bars are mean ± SEM of 6-7 oocytes.

hP2X7 -------------MPACC--SCSDVFQYETNKVTRIQSMNYGTIKWFFHVIIFSYV-CFA 44

hP2X1 ------------MARRFQEELAAFLFEYDTPRMVLVRNKKVGVIFRLIQLVVLVYVIGWV 48

hP2X2 MAAAQPKYPAGATARRLARGCWSALWDYETPKVIVVRNRRLGVLYRAVQLLILLYFVWYV 60

hP2X3 ------------------MNCISDFFTYETTKSVVVKSWTIGIINRVVQLLIISYFVGWV 42

hP2X4 -------------MAGCCAALAAFLFEYDTPRIVLIRSRKVGLMNRAVQLLILAYVIGWV 47

hP2X5 ------------MGQAGCKGLCLSLFDYKTEKYVIAKNKKVGLLYRLLQASILAYLVVWV 48

hP2X6 --MCPQLAGAGSMGSP-GATTGWGLLDYKTEKYVMTRNWRVGALQRLLQFGIVVYVVGWA 57

. *.* : :. * : .: :. *. :.

hP2X7 LVSDKLYQRKEP-VISSVHTKVKGIAEVKEEIVENGVKKLVHSVFDTADYTFPLQG-NSF 102

hP2X1 FLYEKGYQTSSG-LISSVSVKLKGLAVTQ-------LPGLGPQVWDVADYVFPAQGDNSF 100

hP2X2 FIVQKSYQESETGPESSIITKVKGITTSE------------HKVWDVEEYVKPPEGGSVF 108

hP2X3 FLHEKAYQVRDTAIESSVVTKVKGSGLYA------------NRVMDVSDYVTPPQGTSVF 90

hP2X4 FVWEKGYQETDSV-VSSVTTKVKGVAVTN-------TSKLGFRIWDVADYVIPAQEENSL 99

hP2X5 FLIKKGYQDVDTSLQSAVITKVKGVAFTN-------TSDLGQRIWDVADYVIPAQGENVF 101

hP2X6 LLAKKGYQERDLEPQFSIITKLKGVSVTQ-------IKELGNRLWDVADFVKPPQGENVF 110

:: .* ** . :: .*:** : *. ::. * : . :

hP2X7 FVMTNFLKTEGQEQRLCPEYPTR-RTLCSSDRGCKKGWMDPQSKGIQTGRCVVYEG-NQK 160

hP2X1 VVMTNFIVTPKQTQGYCAEHPE--GGICKEDSGCTPGKAKRKAQGIRTGKCVAFND-TVK 157

hP2X2 SIITRVEATHSQTQGTCPESIRVHNATCLSDADCVAGELDMLGNGLRTGRCVPYYQGPSK 168

hP2X3 VIITKMIVTENQMQGFCPESEE--KYRCVSDSQCG--PERLPGGGILTGRCVNYS-SVLR 145

hP2X4 FVMTNVILTMNQTQGLCPEIPDA-TTVCKSDASCTAGSAGTHSNGVSTGRCVAFNG-SVK 157

hP2X5 FVVTNLIVTPNQRQNVCAENEGIPDGACSKDSDCHAGEAVTAGNGVKTGRCLRRENLARG 161

hP2X6 FLVTNFLVTPAQVQGRCPEHPSVPLANCWVDEDCPEGEGGTHSHGVKTGQCVVFNG-THR 169

::*.. * * * * * * * * . *: **:*:

hP2X7 TCEVSAWCPIEAVEEAPRPALLNSAENFTVLIKNNIDFPGHNYTTRNILPGLN----ITC 216

hP2X1 TCEIFGWCPVEVDDDIPRPALLREAENFTLFIKNSISFPRFKVNRRNLVEEVNAAHMKTC 217

hP2X2 TCEVFGWCPVEDGA-SVSQFLGTMAPNFTILIKNSIHYPKFHFSKGNIADR-TDGYLKRC 226

hP2X3 TCEIQGWCPTEVDT-VET-PIMMEAENFTIFIKNSIRFPLFNFEKGNLLPNLTARDMKTC 203

hP2X4 TCEVAAWCPVEDDTHVPQPAFLKAAENFTLLVKNNIWYPKFNFSKRNILPNITTTYLKSC 217

hP2X5 TCEIFAWCPLETSS-RPEEPFLKEAEDFTIFIKNHIRFPKFNFSKSNVMDVKDRSFLKSC 220

hP2X6 TCEIWSWCPVESGV-VPSRPLLAQAQNFTLFIKNTVTFSKFNFSKSNALETWDPTYFKHC 228

***: .*** * : * :**:::** : : .: * *

hP2X7 TFHKTQNPQCPIFRLGDIFRETGDNFSDVAIQGGIMGIEIYWDCNLDRWFHHCRPKYSFR 276

hP2X1 LFHKTLHPLCPVFQLGYVVQESGQNFSTLAEKGGVVGITIDWHCDLDWHVRHCRPIYEFH 277

hP2X2 TFHEASDLYCPIFKLGFIVEKAGESFTELAHKGGVIGVIINWDCDLDLPASECNPKYSFR 286

hP2X3 RFHPDKDPFCPILRVGDVVKFAGQDFAKLARTGGVLGIKIGWVCDLDKAWDQCIPKYSFT 263

hP2X4 IYDAKTDPFCPIFRLGKIVENAGHSFQDMAVEGGIMGIQVNWDCNLDRAASLCLPRYSFR 277

hP2X5 HFGPK-NHYCPIFRLGSVIRWAGSDFQDIALEGGVIGINIEWNCDLDKAASECHPHYSFS 279

hP2X6 RYEPQFSPYCPVFRIGDLVAKAGGTFEDLALLGGSVGIRVHWDCDLDTGDSGCWPHYSFQ 288

: **::::* :. :* * :* ** :*: : * *:** * * *.*

hP2X7 RLDDKTTNVSLYPGYNFRYAKYYKE-NNVEKRTLIKVFGIRFDILVFGTGGKFDIIQLVV 335

hP2X1 GLYE---EKNLSPGFNFRFARHFVE-NGTNYRHLFKVFGIRFDILVDGKAGKFDIIPTMT 333

hP2X2 RLDPKH--VPASSGYNFRFAKYYKI-NGTTTRTLIKAYGIRIDVIVHGQAGKFSLIPTII 343

hP2X3 RLDSVSEKSSVSPGYNFRFAKYYKMENGSEYRTLLKAFGIRFDVLVYGNAGKFNIIPTII 323

hP2X4 RLDTRDVEHNVSPGYNFRFAKYYRDLAGNEQRTLIKAYGIRFDIIVFGKAGKFDIIPTMI 337

hP2X5 RLDNK-LSKSVSSGYNFRFARYYRDAAGVEFRTLMKAYGIRFDVMVNGKAGKFSIIPTII 338

hP2X6 LQE---------KSYNFRTATHWWEQPGVEARTLLKLYGIRFDILVTGQAGKFGLIPTAV 339

.:*** * :: . * *:* :***:*::* * .***.:*

hP2X7 YIGSTLSYFGLAAVFIDFLIDTYSSNCCRSHIYPWCKCCQPCVVNEYYYRKKCESIVEPK 395/595

hP2X1 TIGSGIGIFGVATVLCDLLLLHIL------------------PKRHYYKQKKFKYAEDMG 375/399

hP2X2 NLATALTSVGVGSFLCDWILLTFM------------------NKNKVYSHKKFDKVCTPS 385/406

hP2X3 SSVAAFTSVGVGTVLCDIILLNFL------------------KGADQYKAKKFEEV*NET*T 365/377

hP2X4 NIGSGLALLGMATVLCDIIVLYCM------------------KKRLYYREKKYKYVEDYE 379/388

hP2X5 NVGSGVALMGAGAFFCDLVLIYLI------------------KKREFYRDKKYEEVRGLE 380/406

hP2X6 TLGTGAAWLGVVTFFCDLLLLYVD------------------REAHFYWRTKYEEAKAPK 381/408

: .* :.: * :: * .* .

**SI Appendix, Fig. S7. Protein sequence alignment of the seven hP2X isoforms.** A protein blast search was performed using the 595-residue long hP2X7A subunit as the query sequence in the UNiProKB/Swiss-Prot database. Since the C-terminal endodomains of the different hP2X subunits show minimal homology, the alignment was terminated at position 395 of the 595 amino acid long hP2X7 protein chain. The conserved ectodomain cysteine residues are highlighted in magenta. In the hP2X7 sequence, the positions of TM1 and TM2 are highlighted in yellow, residue S342 is highlighted in turquoise as the gate and acidic residues E14, D352 and D356 are highlighted in red as ion selectivity candidates. Note that E14 of hP2X7 is conserved in all other P2X subunits except hP2X5 and hP2X6, which have a lysine (K, highlighted in blue) at the corresponding position. The N-glycosylation sequons (NXS/T) are highlighted in gray; one sequon, ^374^NET in hP2X3, is intracellular and therefore unused, as indicated in italics. The UniProt/SwissProt accession numbers are P51575.1 (hP2X1); Q9UBL9.1 (hP2X2); P56373.2 (hP2X3); Q99571.2 (hP2X4); Q93086.4 (hP2X5); O15547.2 (hP2X6); Q99572.4 (hP2X7). Since the UniProt/SwissProt only contains the prevailing, but non-functional splice variant, which is missing 22 codons in the pre-TM2 and TM2 regions (Duckwitz et al 2006), the full-length sequence was completed with the missing residues (highlighted in green).

*W. Duckwitz, R. Hausmann, A. Aschrafi, G. Schmalzing*. P2X5 subunit assembly requires scaffolding by the second transmembrane domain and a conserved aspartate. *J Biol Chem* **281**, 39561-39572 (2006).
