# Supplementary material for: Two serial filters control P2X7 cation selectivity, Ser342 in the central pore and lateral acidic residues at the cytoplasmic interface
Source: PNAS Nexus. 2024 Aug 23;3(9):pgae349. doi: 10.1093/pnasnexus/pgae349 (PMC11388005; doi:10.1093/pnasnexus/pgae349)
Supplement: pgae349_Supplementary_Data [file pgae349_supplementary_data.zip › PNASNEXUS-PNASNEXUS-2024-00360-TR-s02.docx]

*SI Appendix*, Table S1. Tris^+^/Na^+^ and Glu^-^/Cl^-^ permeability ratios of wt and mutant hP2X7Rs

| hP2X7R construct | Group in Fig. 3 | Tris^+^/Na^+^ | SEM | N | Glu^-^/Cl^-^ | SEM | N |
| --- | --- | --- | --- | --- | --- | --- | --- |
| wt | 1 | 0.15 | 0.01 | 6 |  |  |  |
|  |  |  |  |  |  |  |  |
| E^14^K | 2 | 0.29 | 0.03 | 6 |  |  |  |
| S^339^A | 2 | 0.30 | 0.01 | 11 |  |  |  |
| S^342^A | 2 | 0.45 | 0.04 | 9 |  |  |  |
| D^352^K | 2 | 0.35 | 0.03 | 6 |  |  |  |
| D^356^K | 2 | 0.27 | 0.01 | 6 |  |  |  |
| E^14^K,D^352^K | 2 | 0.41 | 0.04 | 6 | 0.59 | 0.05 | 6 |
| E^14^K,D^356^K | 2 | 0.40 | 0.04 | 6 | 0.53 | 0.05 | 6 |
| E^14^K,D^352^K,D^356^K | 2 | 0.29 | 0.02 | 6 | 0.68 | 0.04 | 6 |
|  |  |  |  |  |  |  |  |
| S^342^K | 3 | 0.52 | 0.04 | 11 |  |  |  |
| E^14^K,S^342^K | 3 | 0.61 | 0.08 | 6 | 0.57 | 0.04 | 6 |
| E^14^K,S^339^A,S^342^K | 3 | 0.63 | 0.04 | 6 | 0.58 | 0.02 | 6 |
| S^342^K,D^356^K | 3 | 0.57 | 0.03 | 7 | 0.67 | 0.08 | 7 |
|  |  |  |  |  |  |  |  |
| S^342^K,D^352^K | 4 |  |  |  | 0.23 | 0.04 | 5 |
| E^14^K,S^342^K,D^352^K | 4 |  |  |  | 0.22 | 0.05 | 6 |
| E^14^K,S^342^K,D^356^K | 4 |  |  |  | 0.31 | 0.04 | 6 |
| E^14^K,S^339^A,S^342^K,D^356^K | 4 |  |  |  | 0.20 | 0.02 | 6 |
| E^14^K,S^339^A,S^342^K,D^352^K,D^356^K | 4 |  |  |  | 0.17 | 0.04 | 6 |

Permeability ratios were calculated using the Goldman-Hodgkin-Katz equation (Hille 1971) from V_rev_ values determined from I-V curves as shown in Fig. 2. Data are mean ± SEM of measurements on N different oocytes. Calculations are based on the mean reversal potentials shown in Fig. 3. The hP2X7R constructs are divided into the same groups as in Fig. 3 and numbered accordingly. Blank fields indicate that the corresponding hP2X7R constructs did not show significant V_rev_ changes after substitution of Na^+^ or Cl^-^ by Tris^+^ or Glu^-^, respectively, due to negligible cation or anion permeability. Since the derived permeability indices are not meaningful, they have been omitted.

B. Hille. The permeability of the sodium channel to organic cations in myelinated nerve. *J Gen* *Physiol* **58**, 599-619 (1971).
