# Supplementary material for: Two serial filters control P2X7 cation selectivity, Ser342 in the central pore and lateral acidic residues at the cytoplasmic interface
Source: PNAS Nexus. 2024 Aug 23;3(9):pgae349. doi: 10.1093/pnasnexus/pgae349 (PMC11388005; doi:10.1093/pnasnexus/pgae349)
Supplement: pgae349_Supplementary_Data [file pgae349_supplementary_data.zip › PNASNEXUS-PNASNEXUS-2024-00360-TR-s03.docx]

Table S2 – Sequences of mutagenesis primers used in this study

| **Mutated hP2X7 residue(s)** | **Primer number** | **Orienta­tion** | **Primer sequence** | **Generated restriction site** |
| --- | --- | --- | --- | --- |
| E^14^K (K-9442) | O-12.325 | forward | CAGTGATGTTTTCCAGTATAAGACCAACAAAGTCACTAG | -3.BsaI |
|  | O-12.325 | reverse | CTAGTGACTTTGTTGGTCTTATACTGGAAAACATCACTG |  |
| S^339^A (K-8153) | O-10.842 | forward | GTTGTGTACATCGGCGCCACCCTCTCCTACTTC | +1.NarI |
|  | O-10.843 | reverse | GAAGTAGGAGAGGGTGGCGCCGATGTACACAAC |  |
| S^342^A (K-7437) | O-9.877 | forward | GTACATCGGCTCAACCCTCGCCTACTTCGGTCTGG | -2.BspMI |
|  | O-9.878 | reverse | GAAGTAGGAGAGGGTGGCGCCGATGTACACAAC |  |
| S^339^A,S^342^K (K-9558) | O-12.455 | forward | GTTGTGTACATCGGCGCCACCCTCAAGTACTTCGG | +3.BsaHI |
|  | O-12.456 | reverse | CCGAAGTACTTGAGGGTGGCGCCGATGTACACAAC |  |
| S^339^K,S^342^K (K-8157) | O-10.852 | forward | CATCGGCAAAACCCTTAAGTACTTCGGTCTGGCC | +1.AflII |
|  | O-10.853 | reverse | GGCCAGACCGAAGTACTTAAGGGTTTTGCCGATG |  |
| S^339^K (K-8156) | O-10.846 | forward | CATTATCCAGCTGGTTGTATACATCGGCAAAACCCTCTCCTACTTC | +2.AccI |
|  | O-10.847 | reverse | GAAGTAGGAGAGGGTTTTGCCGATGTATACAACCAGCTGGATAATG |  |
| S^342^K (K-7439) | O-9.881 | forward | CATCGGCTCAACCCTCAAGTACTTCGGTCTGGCC | +3.ScaI |
|  | O-9.882 | reverse | GGCCAGACCGAAGTACTTGAGGGTTGAGCCGATG |  |
| S^342^K,Y^343^K (K-8308) | O-11.028 | forward | GGCTCAACCCTCAAGAAATTCGGTCTGGCCGC | -3.ScaI |
|  | O-11.029 | reverse | GCGGCCAGACCGAATTTCTTGAGGGTTGAGCC |  |
| Y^343^K (K-9553) | O-12.453 | forward | GGCTCAACCCTCTCCAAATTCGGTCTGGCCGC | +4.XcmI |
|  | O-12.454 | reverse | GCGGCCAGACCGAATTTGGAGAGGGTTGAGCC |  |
| D^352^K (K-9620) | O-12.529 | forward | GCCGCTGTGTTCATCAAATTCCTCATCGACACTTAC | +6.AcsI |
|  | O-12.530 | reverse | GTAAGTGTCGATGAGGAATTTGATGAACACAGCGGC |  |
| D^356^K (K-9607) | O-12.516 | forward | CATCGACTTCCTCATCAAAACTTATTCCAGTAACTGCTGTC | -4.BpmI |
|  | O-12.517 | reverse | GACAGCAGTTACTGGAATAAGTTTTGATGAGGAAGTCGATG |  |
| D^352^K,D^356^K (K-9677) | O-12.566 | forward | CATCAAATTCCTCATCAAGACTTACAGCAGTAACTGCTGTC | -4.BpmI |
|  | O-12.567 | reverse | GACAGCAGTTACTGCTGTAAGTCTTGATGAGGAATTTGATG |  |

Construct numbers (with 'K- ' prefix) and oligonucleotide numbers (with 'O- ' prefix) refer to internal documentation lists and are used for identification in the laboratory when needed. Nucleotides highlighted in red generate the desired mutation and add or remove the indicated silent restriction site used for initial screening prior to verification by DNA sequencing. The other constructs could be generated from this toolbox of oligonucleotides.
